# EGR2 Deletion Suppresses Anti-DsDNA Autoantibody and IL-17 Production in Autoimmune-Prone B6/lpr Mice: A Differential Immune Regulatory Role of EGR2 in B6/lpr Versus Normal B6 Mice

**DOI:** 10.3389/fimmu.2022.917866

**Published:** 2022-06-15

**Authors:** Rujuan Dai, Zhuang Wang, Bettina Heid, Kristin Eden, Christopher M. Reilly, S. Ansar Ahmed

**Affiliations:** ^1^ Department of Biomedical Sciences and Pathobiology, Virginia-Maryland College of Veterinary Medicine (VMCVM), Virginia Tech, Blacksburg, VA, United States; ^2^ Department of Basic Science Education, Virginia Tech Carilion School of Medicine, Roanoke, VA, United States; ^3^ Department of Biomedical Sciences, Edward Via College of Osteopathic Medicine, Blacksburg, VA, United States

**Keywords:** EGR2, anti-dsDNA autoantibody, IL-17, double negative T cell, plasma cell, murine model

## Abstract

Previous studies have reported that deletion of the transcription factor, early growth response protein 2 (EGR2), in normal C57BL/6 (B6) resulted in the development of lupus-like autoimmune disease. However, increased EGR2 expression has been noted in human and murine lupus, which challenges the notion of the autoimmune suppressive role of EGR2 in B6 mice. In this study, we derived both conditional EGR2^-/-^B6/*lpr* and EGR2^-/-^B6 mice to elucidate the immune and autoimmune regulatory roles of EGR2 in autoinflammation (B6/lpr) versus physiologically normal (B6) conditions. We found that conditional EGR2 deletion increased spleen weight, enhanced T cell activation and IFNγ production, and promoted germinal center B cells and LAG3^+^ regulatory T cells development in both B6/lpr and B6 mice. Nevertheless, EGR2 deletion also showed strikingly differential effects in these two strains on T lymphocyte subsets profile, Foxp3^+^ Tregs and plasma cell differentiation, anti-dsDNA autoantibodies and immunoglobulins production, and on the induction of IL-17 in *in vitro* activated splenocytes. Specifically, EGR2 deletion in B6/lpr mice significantly decreased serum levels of anti-dsDNA autoantibodies, total IgG, IgM, IgG1, and IgG2a with reduced plasma cells differentiation. Furthermore, EGR2 deletion in B6/lpr mice had no obvious effect on IgG immunocomplex deposition, medium caliber vessel, and glomeruli inflammation but increased complement C3 immunocomplex deposition and large caliber vessel inflammation in the kidneys. Importantly, we demonstrated that EGR2 deletion in B6/lpr mice significantly reduced pathogenic CD4^-^CD8^-^CD3^+^B220^+^ double negative T cells, which correlated with the reduced anti-dsDNA autoantibodies in serum and decreased IL-17 production in splenocytes of EGR2^-/-^B6/lpr mice. Together, our data strongly suggest that the role of EGR2 is complex. The immunoregulatory role of EGR2 varies at normal or autoinflammation conditions and should not be generalized in differential experimental settings.

## Introduction

The early growth response protein 2 (EGR2) is a member of Egr zinc finger transcription factor family. EGR2 was initially identified to play a critical role in early hindbrain development and in controlling the process of myelination (1, 2). Deletion of EGR2 led to perinatal death in mice due to major hindbrain defects (1). More recent studies have revealed a vital role of EGR2 in immune system development and function (3–7). In normal C57BL/6 (B6) mice, EGR2 is critically involved in the induction of T cell anergy and EGR2 negatively regulates T cell activation and effector T cell responses (8–10). Conditional deletion of EGR2 in CD2^+^ lymphocytes in non-autoimmune B6 mice led to a break in immune tolerance and induction of a T cell-driven, lupus-like autoimmune disease in EGR2^-/-^B6 mice (10). When compared to control B6 mice, these EGR2^-/-^B6 mice showed heightened T cell activation with the accumulation of CD44^+^ effector CD4^+^ T cells, increased production of inflammatory cytokine IFNγ and IL-17, and significantly higher serum levels of anti-dsDNA autoantibodies and total IgG (10). Further, the B6 mice with conditional deletion of EGR2 in CD4^+^ T cells had enhanced germinal center (GC) response and humoral response (11, 12). This phenotype was attributed to the loss of immune suppressive function of a specific type of Tregs, which are Foxp3 independent and characterized as CD4^+^CD25^-^LAG3^+^ (11, 12).

The studies with conditional EGR2^-/-^B6 mice suggest an autoimmune suppressive role of EGR2 in normal B6 mice (10–13). Nevertheless, a candidate gene promoter polymorphism analysis indicated that increased EGR2 gene expression is linked with lupus susceptibility in humans (14). Elevated EGR2 expression has also been noted in biopsy specimens of skin and lungs from patients with systemic sclerosis, an autoimmune disease that has overlapping symptoms with lupus (15). We recently reported a significant upregulation of EGR2 expression in both human lupus peripheral blood mononuclear cells (PBMCs) and murine lupus T cells (16). Moreover, *in vitro* inhibition of EGR2 with siRNA significantly reduced IFNγ production in activated splenic CD4^+^ T cells from the spontaneously autoimmune lupus-prone MRL/lpr mice, but not control MRL mice (16). These *in vitro* studies suggest that EGR2 may play a differential role in the regulation of inflammatory responses and cytokine production in CD4^+^ T cells of MRL/lpr mice versus CD4^+^ T cells from their relative control MRL mice.

In this study, to further clarify the role of EGR2 in autoinflammation condition versus normal physiological condition, we derived conditional EGR2 knockout mice in both B6/lpr (a lymphoproliferative, systemic lupus erythematosus (SLE)-like autoimmune disease model) and normal B6 genetic background. This comprehensive study shows the comparative and differential immunological, developmental, and functional consequences of EGR2 deletion in autoimmune-prone B6/*lpr* and normal B6.

## Material and Method

### Mice

The Institutional Animal Care and Use Committee (IACUC) of Virginia Tech approved all experimental animal procedures, housing, and care. The B6/lpr (B6.MRL-Fas^lpr^/J, Stock No. 000482), B6 (C57BL/6J, Stock No. 000664), and hCD2-iCre (B6-Cg-Tg(CD2-icre)4Kio/J, Stock No. 008520) mice were purchased from the Jackson Laboratory (JAX, Maine, USA) and maintained in-house. The EGR2^fl/fl^ mice in B6 genetic background were kindly provided by Dr. Warren J. Leonard from NIH/NHLBI. The development and characterization of EGR2^fl/fl^ and EGR2^-/-^ B6 mice have been described in detail in a previous report (6).

The conditional CD2-specific EGR2^-/-^B6 mice were generated by crossbreeding the EGR2^fl/fl^ with hCD2-iCre in the B6 genetic background. To generate conditional CD2-specific EGR2^-/-^B6/lpr mice, EGR2^fl/fl^ and hCD2-iCre mice were crossbred with B6/lpr mice respectively, to obtain EGR2^fl/fl^B6/lpr and CD2-CreB6/lpr strains. Then, the EGR2^fl/fl^B6/lpr and CD2-CreB6/lpr were crossbred to derive CD2-CreEGR2^fl/fl^B6/lpr (referred as EGR2^-/-^B6/lpr) mice. The EGR2^-/-^B6/lpr and EGR2^-/-^B6 mice were bred with EGR2^fl/fl^B6/lpr and EGR2^fl/fl^ B6 mice respectively, to obtain experimental EGR^-/-^B6/lpr and EGR2^-/-^B6 mice. The littermate EGR2^fl/fl^B6/lpr and EGR2^fl/fl^ B6, which do not carry the hCD2-iCre gene, were served as wild-type controls for EGR^-/-^B6/lpr and EGR2^-/-^B6, respectively. Both male and female mice were included in all the experimental procedures in this study. The floxed and deleted EGR2 gene was PCR confirmed as previously reported (6). The presence of hCD2-iCre gene and *lpr* gene was confirmed by following the PCR genotyping protocols provided by the JAX laboratory.

All mice were housed in our Association for Assessment and Accreditation of Laboratory Animal Care (AAALAC)-certified animal facility at the Virginia-Maryland College of Veterinary Medicine (VMCVM), Virginia Tech. Mice were fed with a commercial 7013 NIH- 31 Modified 6% Mouse/Rat Sterilizable Diet (Harlan Laboratory, Madison, WI, USA) and given water *ad libitum*. Compared to MRL/lpr mice, the disease progression in B6/lpr mice is slow and less severe; and B6/lpr mice have a life span of more than 7 months (17, 18). We, therefore, euthanized the EGR2^-/-^B6/lpr and littermate control EGR2^fl/fl^B6/lpr mice at 6-7 months of age to investigate the effects of EGR2 deletion in B6/lpr mice. Since the previous study showed that EGR^-/-^B6 mice did not manifest lupus-like phenotypes until 6-8 months of age (10), we euthanized EGR2^-/-^B6 and littermate control EGR2^fl/fl^B6 mice at 9-10 months of age to investigate the immunoregulatory roles of EGR2 in B6 mice in this study. All the mice were euthanized by CO_2_ asphyxiation followed by either cervical dislocation or heart puncture (for blood collection) in accordance with the IACUC approved protocol.

### Cell Preparation and Culture

The spleen tissue was dissociated into a single cell suspension following a standard laboratory procedure (19–21). The splenocytes were resuspended at a concentration of 5x10^6^/ml in complete RPMI-1640 medium (Hyclone, Logan, UT, USA) supplemented with 10% heat-inactivated fetal bovine serum (Atlanta Biologicals, Flowery Branch, GA, USA), 1% essential amino acids (Hyclone), 2mM L-glutamine (Hyclone), 100IU/mL penicillin/100μg/mL streptomycin (Hyclone) for cell culture. The splenocytes (2.5x10^6^) were plated in 24 well plate and stimulated with phorbol 12-myristate 13-acetate (PMA, 50 ng/ml, Sigma-Aldrich, St. Louis, MO, USA) plus ionomycin (1μg/ml, Sigma-Aldrich) without or with protein transport inhibitor (BD GolgiPlug) for 6 hours to analyze cytokines expression. For the induction of IL-17, the splenocytes (1.25x10^6^) were plated in 48 well plate and stimulated with either non-pathogenic T helper 17 (Th17) stimuli (20 ng/mL recombinant mouse IL-6 (BioLegend, San Diego, CA, USA) plus 3ng/mL recombinant human TGFβ1 (R&D System Inc., Minneapolis, MN, USA) and 1μg/mL soluble anti-CD3) or pathogenic Th17 stimuli (20 ng/mL recombinant mouse IL-6, 10 ng/mL recombinant mouse IL-1β (BioLegend) plus 10ng/mL recombinant mouse IL-23 (eBioscience/Thermo Fisher Scientific, Carlsbad, CA, USA) and 1μg/mL soluble anti-CD3) for 72 hours. The cell culture supernatants were collected for ELISA assay.

Bone marrow cells were prepared by flushing the mouse femora and tibia with ice-cold PBS. The flushed-out cells were passed through a 70 μM cell strainer, pelleted, resuspended with PBS, and then treated with Ammonium-Chloride-Potassium (ACK) lysis buffer to remove red blood cells. The isolated bone marrow cells were resuspended in MACS buffer (PBS supplemented with 0.5% BSA and 2mM EDTA) for flow cytometry analysis.

### Flow Cytometry

Flow cytometric analysis was performed to determine specific immune cell subsets in the spleen and bone marrow as we have previously reported (16, 20). The fluorochrome‐conjugated antibodies used for staining cell surface markers included: efluor-450 (e450)-CD4 (clone RM4-5), APC-CD8 (clone 53-6.7), FITC-CD3ε (clone 145-2C11), PE-B220 (clone RA3-6B2), PerCP/Cy5.5-CD19 (clone eBio1D3), PerCP/Cy5.5-CD44 (clone IM7), PE/Cy7-CD62L (clone MEL-14), PerCP/Cy5.5-CD25 (clone PC61.5), e660-GL7 (clone GL-7), e450-CD23 (clone B3B4), and PE-CD223 (LAG3, clone eBioC9B7W) from eBioscience; APC-CD69 (clone H1.2F3), FITC-CD21/35 (clone 7G6) and PE/Cy7-CD43 (clone S7) from BD Bioscience, Franklin Lakes, NJ, USA; APC/Cy7-IgD (clone 11-26c.2a), PE-CD138 (clone 281-2), and APC-IgM (clone RMM-1) from BioLegend. Dead cells were excluded for the analysis by staining the cells with propidium iodide (PI, from ImmunoChemistry Technology, LLC, MN, USA). The Foxp3 transcription factor staining buffer set (eBioscience) was used for flow cytometry analysis of intracellular antigens as we previously reported (16). PE-EGR2 (clone erongr2), Percp-cy5.5-IFNγ (XMG1.2) and FITC-IL-17A (eBio17B7) were obtained from eBioscience. Alexa-fluor 647 (AF647)-Foxp3 (clone MF-14) was purchased from BioLegend. Flow-stained cell samples were analyzed on a FACSAria Flow cytometer (BD Biosciences). The data were analyzed with FlowJo software (FlowJo, LLC, Ashland, OR, USA). The cells were first gated to remove cell debris (SSC-A vs. FSC-A), then gated for singlets (FSC-H vs. FSC-W). Except for flow analysis of the intracellular antigens, the PI negative (live cells) were further gated to profile the immune cell subsets.

### Th1 and Th17 Cell Differentiation

Naïve CD4^+^ T cells were purified from the splenocytes of 6-7-week-old EGR2^-/-^B6/lpr and control mice by using a mouse naïve CD4^+^ T cells isolation kit from Miltenyi Biotec as we previously reported (16). For Th1 differentiation, naïve CD4^+^ T cells (1.5x10^6^) were plated in 48-well plate and cultured with 2 μg/ml plate-bound anti-CD3 (clone 145-2C11, Bio X cell, Lebanon, NH, USA), 1 μg/ml soluble anti-CD28 (clone 37.51, Bio X cell), 5ng/ml IL-2 (eBioscience), 10ng/ml IL-12 (eBioscience) and 10 μg/ml anti-IL4 (Bio X cell) for 3 days. For Th17 differentiation, naïve CD4^+^ T cells (1.5x10^6^) were plated in 48-well plate and cultured with 2 μg/ml plate-bound anti-CD3, 1 μg/ml soluble anti-CD28, 20ng/ml IL-6, 3ng/ml hTGFβ1, 10 μg/ml anti-IFNγ (Bio X cell) and 10 μg/ml anti-IL4 for 5 days. The differentiated cells were stimulated with PMA (50 ng/ml), ionomycin (1 μg/ml) plus protein transporter inhibitor (BD GolgiPlug) for 5 hours, and then collected for intracellular flow cytometry analysis of IFNγ- and IL-17- expressing cells.

### ELISA

The levels of cytokines in culture supernatant were determined by ELISA kits from Biolegend and ThermoFisher Scientific. A laboratory standardized ELISA procedure was performed to determine the serum level of anti-dsDNA autoantibody (22–24). Serum levels of IgG, IgM, IgG1, and IgG2a were quantified with the specific ELISA kits (ThermoFisher Scientific) by following manufactory instructions.

### Serum Blood Urea Nitrogen Measurement

The serum Blood Urea Nitrogen (BUN) levels were measured with a BUN Colorimetric Detection kit (ThermoFisher Scientific) to evaluate the renal function change.

### Renal Histopathology

Renal histopathology analysis was performed as we previously reported (21–23). Briefly, the kidneys were fixed with 10% buffered formalin, embedded in paraffin, sectioned to 5μM, and then stained with hematoxylin and eosin (H&E) in the ViTALS laboratory at VMCVM, Virginia Tech. A board-certified veterinary pathologist assessed the H&E-stained renal sections in a blinded fashion. For inflammation, scattered and infrequent deposits of lymphocytes, plasma cells, macrophages, and/or neutrophils distributed in a nonspecific fashion were scored as a 1. Denser deposits targeting identifiable vascular structures were scored as a 2, and large deposits that effaced the local renal parenchyma and vasculature scored as a 3. Inflammation locations were divided into the renal pelvis/renal artery/interlobar (large caliber vessel) and arcuate/interlobular (medium caliber vessel). Glomerular changes were scored by evaluating mesangial cellularity, mesangial thickening/matrix deposition, average glomerular size, and presence of inflammatory cells, with a score of 1 indicating mild, 2 indicating moderate, and 3 indicating severe.

### Renal Immunofluorescence

Kidney sections were possessed as we have previously reported (21, 23). Briefly, kidneys were cut in half, immersed in Tissue-Tek O.C.T. Compound (Sakura Finetek, Torrance, CA, USA) and snap freeze in a dry ice bath containing isopropanol. OCT embed frozen kidneys were cut into 5um sections in a cryostat, mounted to histological slides in the ViTALS lab at VMCVM. The frozen kidney section slides were air-dried, fixed with acetone, blocked with anti-CD16/32 (eBiosceince) in PBS with 1% BSA, and then stained with FITC-conjugated rat anti-mouse IgG (H+L) (eBioscience) or PE-conjugated rat anti-mouse complement C3 (clone RmC11H9; CEDARLANE, Burlington, Ontario, Canada). The stained slides were mounted with coverslips using Prolong Gold anti-fade reagent (ThermoFisher Scientific) and imaged with a digital fluorescence microscope EVOS M5000 (ThermoFisher Scientific). The fluorescent intensities of deposited IgG and C3 were measured with Fiji/ImageJ processing program and normalized by subtracting the background fluorescence intensity (25). At least six glomeruli were analyzed per sample.

### Statistical Analysis

The graphic presentations of data were generated with GraphPad Prism software. All values in graphs were given as means ± SD. Two-tailed, unpaired parametric *t*-tests embedded in the Prism software were performed to determine the statistical significance between control and conditional EGR2 knockout mice (EGR2^fl/fl^B6/lpr vs. EGR2^-/-^B6/lpr; EGR2^fl/fl^B6 vs. EGR2^-/-^B6). *, **, ***, and **** indicate *p* < 0.05, *p* < 0.01, *p* < 0.001, and p<0.0001, respectively.

## Results

### EGR2 Deletion in B6/lpr Mice Promotes Splenomegaly but Reduces Serum Levels of Anti-dsDNA Autoantibodies and Total Immunoglobins

**Figure 1:**
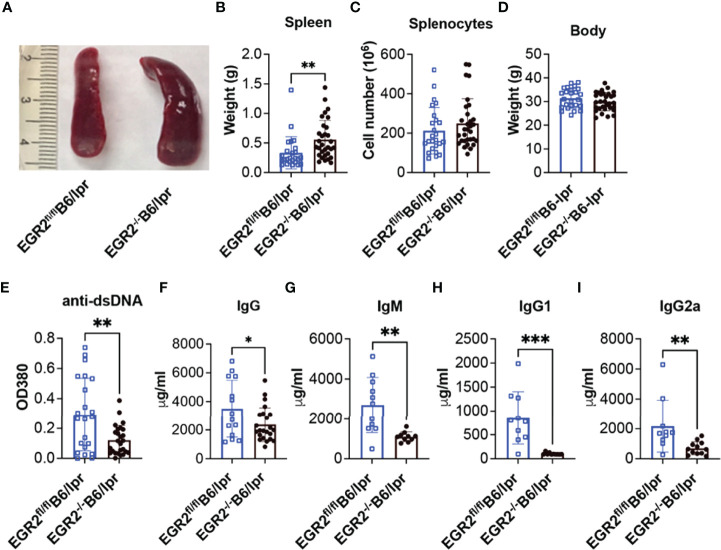
EGR2 deletion in B6/lpr mice promotes splenomegaly but reduces anti-dsDNA autoantibody and total immunoglobins in serum. **(A–D)** Representative spleen images **(A)** and summary graph of spleen weight **(B)**, total splenocytes number **(C)** and body weight **(D)** of 6-7-month-old EGR2^-/-^B6/lpr (n=31) and littermate control EGR2^fl/fl^B6/lpr mice (n=25). **(E–I)** Summary graphs show significantly reduced anti-dsDNA autoantibody (**A**, n≥22), total IgG (**B**, n≥14), total IgM (**C**, n ≥10), IgG1(**D**, n ≥10) and IgG2a (**E**, n≥10) in the serums of EGR2^-/-^B6/lpr mice compared to control EGR2^fl/fl^B6/lpr mice. Horizontal bars in the summary graphs indicated mean ± SD. Statistical significance was indicated by asterisks: *p<0.05; **p<0.01; ***P<0.001.

Since EGR2 null mice are perinatally lethal (1, 2), we used the hCD2-iCre transgenic line (26), which deletes the floxed target gene in both T and B lymphocytes to generate a conditional EGR2 knockout B6/lpr mouse model. We verified the deletion of EGR2 in CD4^+^ T, CD8^+^ T, and CD19^+^ B cells of EGR2^-/-^B6/lpr mice (**Supplemental Figure 1**). Consistent with the previous report that EGR2 deletion promoted splenomegaly in B6 mice (10), we found that EGR2 deletion in B6/lpr mice also significantly increased spleen weight (**Figures 1A, B**). However, there was no significant difference in the absolute splenic cell numbers between EGR2^-/-^B6/lpr and littermate control EGR2^fl/fl^B6/lpr mice (**Figure 1C**). EGR2 deletion did not affect the bodyweight of B6/lpr mice at the endpoint (**Figure 1D**).

It has been reported that EGR2 deletion in B6 mice induced lupus-like autoimmune disease in aged mice with increased levels of anti-dsDNA autoantibodies and total IgG in serum (10). In striking contrast to B6 mice, EGR2 deletion in B6/lpr mice significantly reduced serum levels of anti-dsDNA autoantibodies, total IgG, IgM, IgG1, and IgG2a subtypes (**Figures 1E–I**).

### EGR2 Deletion in B6/lpr Mice Has Limited Effects on Renal Function

**Figure 2:**
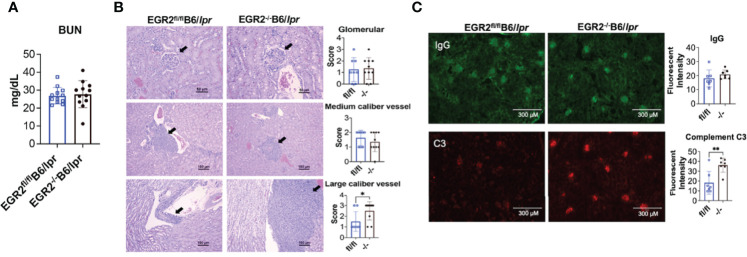
EGR2 deletion in B6/lpr mice increases large caliber vessel inflammation and complement C3 immunocomplex deposition in the kidneys but has no obvious effect on renal function. **(A)** The summary graph shows no difference in the serum levels of blood urea nitrogen (BUN) between EGR2^-/-^B6/lpr and littermate control EGR2^fl/fl^B6/lpr mice (n=12 each). **(B)** Renal histopathology data show that EGR2 deletion only increased inflammation in large caliber vessels, but not in medium caliber vessels and glomeruli. Representative H&E-stained images that reflect the inflammation and pathological changes at glomeruli (top, representing score 2 images for both control fl/fl and knock out -/- mice), medium caliber vessels (middle; representing score 2 images for both fl/fl and -/- mice), and the large caliber vessels (bottom, representing score 1 image for fl/fl and score 3 image for -/- mice) were shown. The summary data are presented on the side (n≥8). **(C)** Representative renal immunofluorescence images and summary graphs show increased complement C3 immunocomplex deposition and no change of IgG immunocomplex deposition in EGR2^-/-^B6/lpr (-/-) compared to control EGR2^fl/fl^B6/lpr (fl/fl) mice (n=7 each). Horizontal bars in the summary graphs indicated mean ± SD. Statistical significance was indicated by asterisks: *p<0.05 and **p<0.01.

A previous study has shown that EGR2^-/-^B6 mice had increased IgG deposition in kidney and developed severe glomerulonephritis with the production of proteinuria (10). We were unable to detect obvious proteinuria production in both EGR2^-/-^B6/lpr and control EGR2^fl/fl^B6/lpr mice. Further, there was no difference in the serum BUN levels between EGR2^-/-^B6/lpr and control EGR2^fl/fl^B6/lpr mice (**Figure 2A**), suggesting EGR2 deletion has no significant effect on renal function in B6/lpr mice. The renal histopathology showed that deleting EGR2 in B6/lpr mice did not enhance glomerular inflammation and changes in B6/lpr mice (**Figure 2B**). We only observed a significant increase of immune cell infiltrating and inflammation in large caliber vessels in renal pelvis/renal artery/interlobar area, but not in medium caliber vessels at the arcuate/interlobular region in the kidneys of EGR2^-/-^B6/lpr mice (**Figure 2B**). EGR2 deletion in B6/lpr mice did not increase IgG immune complexes deposition in the kidneys (**Figure 2C**). However, compared to controls, there was a significant increase of complement C3 immune complexes deposition in the kidneys of EGR2^-/-^ B6/lpr (**Figure 2C**). Together, our data show that EGR2 deletion in B6/lpr mice only increased complement C3 immune complexes deposition and large vascular inflammation in kidney and it had no significant effects on IgG deposition, glomerular inflammation, and renal function.

### EGR2 Deletion in B6 Mice Promotes Splenomegaly and Anti-dsDNA Autoantibodies Production

**Figure 3:**
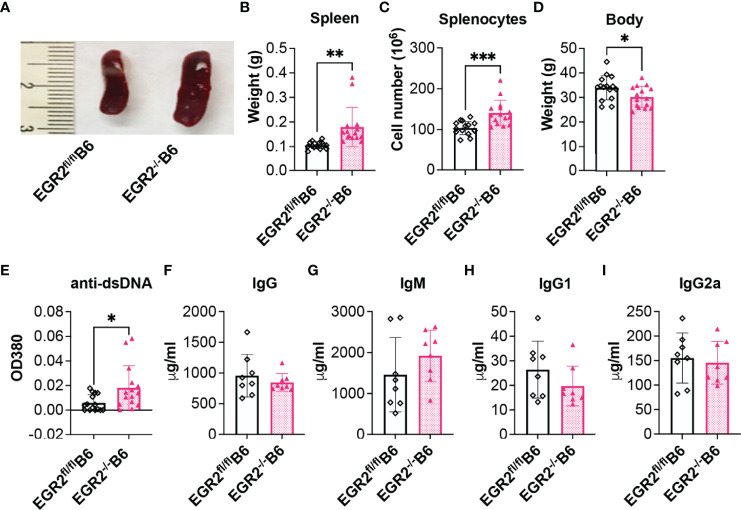
EGR2 deletion in B6 mice promotes splenomegaly and anti-dsDNA autoantibody production. **(A–D)** Representative spleen images **(A)** and summary graph of spleen weight **(B)**, splenocytes number **(C)**, and body weight **(D)** of 9-10-month-old EGR2^-/-^B6 mice and littermate control EGR2^fl/fl^B6 mice (n=15 each). **(E–I)** Summary graphs show significantly increased anti-dsDNA autoantibody (**E**, n=15 each) and no obvious changes in total IgG (**G**, n=8 each), total IgM (**H**, n=8 each), IgG1(**I**, n=8 each) and IgG2a (**J**, n=8 each) in the serums of EGR2^-/-^B6 mice compared to control EGR2^fl/fl^B6 mice. Horizontal bars in the summary graphs indicated mean ± SD. Statistical significance was indicated by asterisks: *p<0.05; **p<0.01; ***P<0.001.

The above data with EGR2^-/-^B6/lpr mice showed that EGR2 deletion reduced serum levels of anti-dsDNA autoantibodies and total IgG, which is inconsistent with the previous report (10). To compare the effect of EGR2 deletion in the context of autoinflammation (B6/lpr) and normal physiological states (B6) at the same experimental settings, we generated conditional EGR2 knockout B6 mice (EGR2^-/-^B6) with the same hCD2-iCre strain in our facility. Consistently with the previous report (10), there was a significant increase in spleen weight and cellularity in EGR2^-/-^B6 mice compared to controls (**Figures 3A–C**). While EGR2 deletion did not affect the bodyweight of B6/lpr mice at the endpoint (**Figure 1D**), it reduced the bodyweight of B6 mice at the endpoint (**Figure 3D**). We found that EGR2 deletion increased serum levels of anti-dsDNA autoantibodies in B6 mice, although in much less magnitude than previously reported (**Figure 3E**). However, we did not notice obvious changes in the serum levels of total IgG, IgM, IgG1, and IgG2a between EGR2^-/-^B6 mice and controls (**Figures 3F–I**). We were also unable to detect proteinuria and immune complex deposition in the kidneys of EGR2^-/-^B6 mice (data not shown). Together, our data with EGR2^-/-^B6/lpr and EGR2^-/-^B6 mice demonstrated that EGR2 has a differential role in the regulation of immunoglobins and anti-dsDNA autoantibody production in B6/lpr versus B6 mice.

### Deletion of EGR2 Suppresses Plasma Cell Differentiation in B6/lpr, but Not in B6 Mice

**Figure 4:**
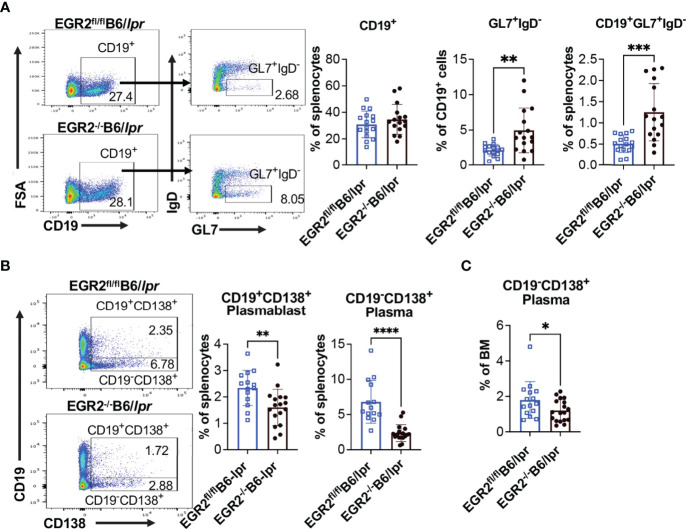
EGR2 deletion in B6/lpr mice promotes GC B cell development but suppresses plasma cell differentiation. **(A)** Representative flow plots and summary graphs show significantly increased percentage of GL7^+^IgD^-^ cells in gated splenic CD19^+^ cells and increased percentage of CD19^+^GL7^+^IgD^-^ cells in the splenocytes of EGR2^-/-^B6/lpr mice compared to controls (n=16 each). **(B)** Representative flow plots and summary graphs show significantly reduced percentage of CD19^+^CD138^+^ plasmablasts and CD19^-^CD138^+^ plasma cells in the spleens of EGR2^-/-^B6/lpr mice compared to control EGR2^fl/fl^B6/lpr mice (n≥14). **(C)** Summary graph show reduced percentage of CD19^-^CD138^+^ plasma cells in the bone marrows of EGR2^-/-^B6/lpr mice compared to controls (n≥15). Horizontal bars in the summary graphs indicated mean ± SD. Statistical significance was indicated by asterisks: *p<0.05; **p<0.01; ***p<0.001; ****P<0.0001.

**Figure 5:**
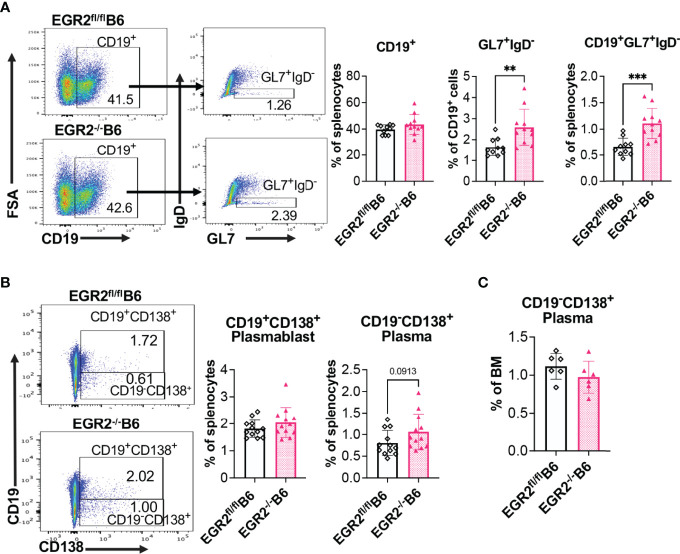
EGR2 deletion in B6 mice promotes GC B cell development but has no significant effect on plasma cell differentiation. **(A)** Representative flow plots and summary graphs show significantly increased percentage of GL7^+^IgD^-^ cells in gated splenic CD19^+^ cells and increased percentage of CD19^+^GL7^+^IgD^-^ cells in the splenocytes of EGR2^-/-^B6 mice compared to controls (n≥10). **(B)** Representative flow plots and summary graphs show no significant difference in the percentage of CD19^+^CD138^+^ plasmablasts and CD19^-^CD138^+^plasma cells in the spleens of EGR2^-/-^B6 mice compared to control EGR2^fl/fl^B6 mice (n=12 each). **(C)** Summary graph show no change of the percentage of CD19^-^CD138^+^ plasma cells in the bone marrows between EGR2^-/-^B6 and control mice (n=6 each). Horizontal bars in the summary graphs indicated mean ± SD. Statistical significance was indicated by asterisks: **p<0.01; ***p<0.001.

To understand the cellular mechanism underlying reduced antibody production in EGR2^-/-^B6/lpr mice, but not in EGR2^-/-^B6 mice, we performed flow cytometry to analyze the development of germinal center (GC) B cells and major antibody-producing cells, plasmablasts and plasma cells. In both B6/lpr and B6 mice, EGR2 deletion did not affect the percentage of CD19^+^ B cells in the spleens but significantly increased GL7^+^IgD^-^ GC B cells in the gated CD19^+^ cells, leading to the increase of percentage CD19^+^GL7^+^IgD^-^ GC B cells in the splenocytes (**Figures 4A**, **5A**). Importantly, we found that EGR2 deletion in B6/lpr mice significantly reduced the percentage of both CD19^+^CD138^+^ plasmablasts and CD19^-^CD138^+^ plasma cells in the spleens (**Figure 4B**). However, in normal B6 mice, EGR2 deletion led to a trend of increase in the percentage of CD19^-^CD138^+^ plasma cells and no change of CD19^+^CD138^+^ plasmablasts in the spleens (**Figure 5B**). We also observed a reduction in the percentage of CD19^-^CD138^+^ plasma cells in the bone marrow cells of EGR2^-/-^B6/lpr, but not in EGR2^-/-^B6 mice (**Figures 4C**, **5C**). Further flow analysis of B cell development in bone marrow indicated that EGR2 deletion did not significantly affect the early B cell development (Pre-ProB, Pro-B, Pre-B, immature/mature B) in both B6/lpr and B6 mice (**Supplemental Figures 2A, B**). Moreover, EGR2 deletion did not significantly affect the development of marginal zone B (MZB) and follicular B (FOB) in the spleens of both B6/lpr and B6 mice (**Supplemental Figures 2C, D**). Together, our data indicate that EGR2 deletion specifically affects the late B cell development/differentiation (GC B and plasma cell) and has no significant effect on early B cell development. EGR2 deletion promotes GCB development in both B6/lpr and B6 mice but apparently has an opposite effect on the differentiation of plasma cells in these two strains.

### EGR2 Deletion Has a Differential Effect on the Development of Atypical CD25^-^LAG3^+^ Tregs and Traditional CD25^+^Foxp3^+^ Tregs in B6/lpr

**Figure 6:**
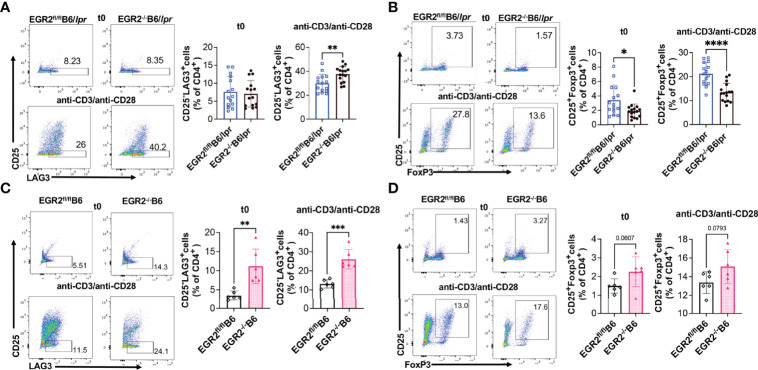
EGR2 deletion in B6/lpr mice has differential effect on the development of LAG3^+^ Tregs and Foxp3^+^ Tregs. The percentage of CD25^-^LAG3^+^ and CD25^+^Foxp3^+^ cells in the gated splenic CD4^+^ T from freshly-isolated (t0) or anti-CD3 plus anti-CD28 stimulated (48 hrs.) splenocytes were analyzed by Flow cytometry. The splenocytes were first gated for CD4^+^ T cells, and then further gated for CD25^-^LAG3^+^ and CD25^+^Foxp3^+^ Tregs. **(A, B)** Representative flow plots show the gating of CD25^-^LAG3^+^ Tregs **(A)** CD25^+^Foxp3^+^ Tregs **(B)** in gated CD4^+^ T cells of fresh or activated splenocytes from EGR2^-/-^B6/lpr mice and control EGR2^fl/fl^B6/lpr (n≥15). **(C, D)** Representative flow plots show the gating of CD25^-^LAG3^+^ Tregs **(C)** CD25^+^Foxp3^+^ Tregs **(D)** in gated CD4^+^ T cells of fresh or activated splenocytes from EGR2^-/-^B6 and control EGR2^fl/fl^B6 mice (n=6 each). Horizontal bars in the summary graphs indicated mean ± SD. Statistical significance was indicated by asterisks: *p<0.05; **p<0.01; ***p<0.001; ****P<0.0001.

A series of studies from Dr. Keishi Fujio’s research group has shown that EGR2 is required for the immune suppressive function of a specific group of LAG3 expressing, Foxp3 independent Tregs (CD4^+^CD25^-^LAG3^+^ Tregs; hereinafter referred to as LAG3^+^ Tregs) (3, 11, 12, 27). EGR2^-/-^B6 and EGR2^-/-^EGR3^-/-^ double knockout B6 mice had enhanced GC response and antibodies production due to the loss of immune suppressive function of LAG3^+^Treg (11, 12). While EGR2 deletion had no obvious effect on the percentage CD25^-^LAG3^+^cells in the gated CD4^+^ T cells of freshly-isolated splenocytes, deleting EGR2 significantly increased the percentage of CD25^-^LAG3^+^ cells in the gated CD4^+^ T cells of anti-CD3 plus anti-CD28 stimulated splenocytes from EGR2^-/-^B6/lpr mice (**Figure 6A**). However, we found that deleting EGR2 in B6/lpr mice significantly reduced the percentage of CD25^+^Foxp3^+^cells in the gated CD4^+^ T cells of both freshly-isolated and anti-CD3 plus anti-CD28 stimulated splenocytes (**Figure 6B**). In B6 mice, EGR2 deletion led to a significant increase of CD25^-^LAG3^+^ cells in gated CD4^+^ cells of both freshly-isolated and anti-CD3 plus anti-CD28 activated splenocytes (**Figure 6C**). These data are consistent with the previous report that EGR2 is dispensable for LAG3^+^ Treg development (11). The previous study did not show a significant effect of EGR2 deletion on Foxp3^+^ Treg in normal B6 mice (6). We also only observed a trend of increase in the percentage of Foxp3^+^CD25^+^ Tregs in gated CD4^+^ cells of either freshly isolated or activated splenocytes from EGR2^-/-^B6 mice (**Figure 6D**). Together, our data demonstrate that EGR2 differentially regulates the development of Foxp3^+^ Tregs and LAG3^+^ Tregs in the B6/lpr mice.

### EGR2 Deletion Increases IFNγ, but Decreases IL-17 Production in Activated Splenocytes of B6/lpr Mice

**Figure 7:**
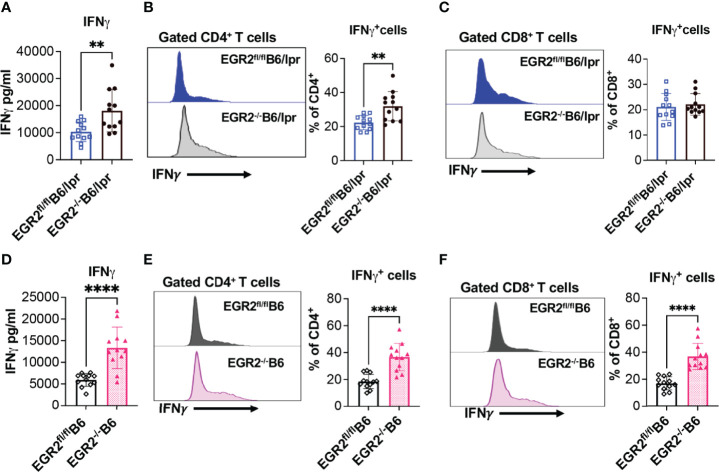
EGR2 deletion promotes IFNγ production in *in vitro* activated splenocytes and splenic CD4^+^ T from either B6/lpr or B6 mice. The splenocytes of EGR2^-/-^B6/lpr, EGR2^-/-^B6 and their respective control EGR2^fl/fl^B6/lpr and EGR2^fl/fl^B6 mice were stimulated with PMA plus ionomycin without **(A, D)** or with protein transport inhibitor **(B, C, E, F)** for 6 hrs. The IFNγ levels in the culture supernatant were measured by ELISA. The IFNγ-expressing CD4^+^ and CD8^+^ T cells were determined by intracellular flow cytometry analysis. **(A)** The summary graph shows an increase of IFNγ production in activated splenocytes from EGR2^-/-^B6/lpr mice compared to control EGR2^fl/fl^B6/lpr mice (n=12 each). **(B, C)** Representative flow histogram and summary graphs show increased percentage of IFNγ-expressing cells in CD4^+^T cells, but not in CD8^+^T cells of EGR2^-/-^B6/lpr mice (n=12 each). **(D)** The summary graph shows an increase of IFNγ production in activated splenocytes from EGR2^-/-^B6 mice compared to control EGR2^fl/fl^B6 mice (n=12 each). **(E, F)** Representative flow histogram and summary graphs show increased percentage of IFNγ-expressing cells in both CD4^+^ T cells and CD8^+^ T cells of EGR2^-/-^B6 mice (n=12 each). Horizontal bars in the summary graphs indicated mean ± SD. Statistical significance was indicated by asterisks: **, p<0.01; ****, P<0.0001.

**Figure 8:**
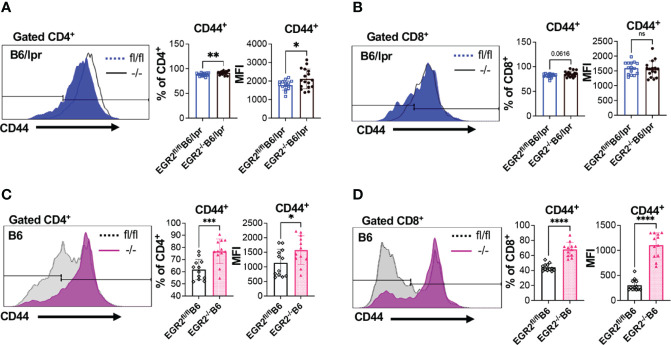
Promotion effect of EGR2 deletion on T cell activation in B6/lpr and B6 mice. **(A, B)** Representative flow histograms and summary graphs show significantly increased CD44^+^ percentage and expression intensity (MFI: mean fluorescence intensity) in gated CD4^+^ T cells **(A)**, but not CD8^+^ T cells **(B)** of freshly-isolated splenocytes of EGR2^-/-^B6/lpr mice (n≥15). **(C, D)** Representative flow histograms and summary graphs show significantly increased CD44^+^ percentage and expression intensity in both gated CD4^+^ T cells **(C)** and CD8^+^ T cells **(D)** of freshly-isolated splenocytes of EGR2^-/-^B6 mice (n=12). Horizontal bars in the summary graphs indicated mean ± SD. Statistical significance was indicated by asterisks: *p<0.05; **p<0.01; ***p<0.001; ****P<0.0001. ns, not statistically significant.

One of the important roles of EGR2 is the negative regulation of T cell activation. Major supportive evidence for the autoimmune suppressive role of EGR2 in B6 mice is that EGR2 deletion induced T cell hyperactivation and heightened production of inflammatory cytokines IFNγ and IL-17 (10). In consistency, we found that deleting EGR2 *in vivo* significantly increased IFNγ production in *in vitro* activated splenocytes from both B6/lpr and B6 mice (**Figures 7A, D**). The intracellular cytokine analysis demonstrated that EGR2 deletion in B6/lpr mice increased IFNγ expression in gated splenic CD4^+^ T cells, but not CD8^+^ T cells (**Figures 7B, C**). Correspondingly, there was a significant increase in the percentage of CD44^+^ cells and CD44 expression intensity in gated CD4^+^ T cells, but not in CD8^+^ T cells of EGR2^-/-^B6/lpr (**Figures 8A, B**). It is noticeable that the majority of the splenic CD4^+^ T and CD8^+^ T cells were already activated and possessed CD44 markers (about 80%) in the B6/lpr mice at the experimental age. EGR2 deletion in B6 mice significantly increased IFNγ expression in both gated splenic CD4^+^ T cells and CD8^+^ T cells (**Figures 7E, F**). Correspondingly, there was a significant increase in the percentage of CD44^+^ cells and CD44 expression intensity in both gated splenic CD4^+^ T and CD8^+^ T cells of EGR2^-/-^B6 mice compared to controls (**Figures 8C, D**).

**Figure 9:**
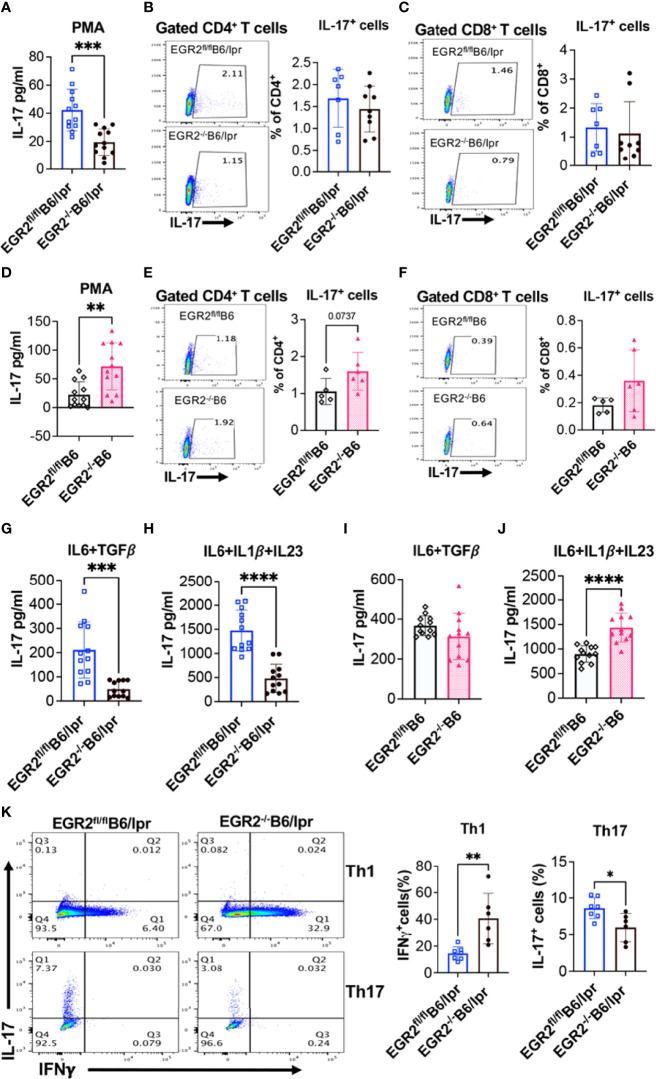
EGR2 deletion has an opposite effect on IL-17 production in activated splenocytes from B6/lpr versus B6 mice. The splenocytes of EGR2^-/-^B6/lpr, EGR2^-/-^B6 and their respective control EGR2^fl/fl^B6/lpr and EGR2^fl/fl^B6 mice were stimulated either with PMA plus ionomycin without **(A, D)** or with protein transport inhibitor **(B, C, E, F)** for 6 hrs, or with non-pathogenic Th17 stimuli (IL6+TGFβ) or pathogenic Th17 stimuli (IL6+IL1β+IL23) for 72 hrs. **(G–J).** The IL-17 levels in the culture supernatant were measured by ELISA. The IL17-expressing CD4^+^ and CD8^+^ T cells were analyzed by intracellular flow cytometry analysis. **(A)** The summary graph shows a reduction of IL-17 production in PMA plus ionomycin activated splenocytes from EGR2^-/-^B6/lpr mice compared to control EGR2^fl/fl^B6/lpr mice (n=12 each). **(B, C)** Representative flow plots and summary graphs show no changes in the percentage of IL-17-expressing cells in CD4^+^T cells and CD8^+^T cells of EGR2^-/-^B6/lpr mice (n≥7). **(D)** The summary graph shows an increase of IL-17 production in PMA plus ionomycin activated splenocytes from EGR2^-/-^B6 mice compared to control EGR2^fl/fl^B6 mice (n=12 each). **(E, F)** Representative flow plots and summary graphs show no significant change in the percentage of IL-17-expressing cells in CD4^+^ T cells and CD8^+^ T cells of EGR2^-/-^B6 mice (n≥5). **(G, H)** Summary graphs show a significant reduction of IL-17 production in either non-pathogenic or pathogenic Th17 stimuli activated splenocytes from EGR2^-/-^B6/lpr mice compared to control EGR2^fl/fl^B6/lpr mice (n=12 each). **(I, J)** Summary graphs show a significant increase of IL-17 production in pathogenic Th17 stimuli but not non-pathogenic Th17 stimuli activated splenocytes from EGR2^-/-^B6 mice control EGR2^fl/fl^B6 (n=12 each). **(K)** Naïve CD4^+^ T cells were differentiated under Th1 condition for 3 days or Th17 condition for 5 days. The expression of IL-17 and IFNγ in Th1 and Th17 differentiated cells were detected by intracellular flow cytometry. The representative flow plots and summary graphs show an increase of IFNγ-expressing cells in Th1 differentiated cells and a significant decrease of IL-17-expressing cells in Th17 differentiated cells from EGR2^-/-^B6/lpr mice (n≥6). Horizontal bars in the summary graphs indicated mean ± SD. Statistical significance was indicated by asterisks: *, p<0.05; **, p<0.01; ***, p<0.001; ****, P<0.0001.

Remarkably, we found that EGR2 deletion had a contradictory effect on IL-17 production in *in vitro* activated splenocytes from B6/lpr versus B6 mice. EGR2 deletion significantly reduced IL-17 production in PMA plus ionomycin activated splenocytes from EGR2^-/-^B6/lpr mice without affecting IL-17 expression in either gated CD4^+^ T or CD8^+^ T cells (**Figures 9A–C**). However, EGR2 deletion in B6 mice significantly increased IL-17 production in PMA plus ionomycin activated splenocytes (**Figure 9D**). The intracellular cytokine analysis showed a trend of increase of IL-17 expression in gated CD4^+^, but not CD8^+^ T cells of EGR2^-/-^B6 mice (**Figures 9E, F**)

We then activated splenocytes with Th17-inducing stimuli to confirm the differential role of EGR2 in the regulation of Th17 effector responses in B6/lpr versus B6 mice. Following stimulation with either non-pathogenic Th17 stimuli (IL-6 plus TGFβ1) or pathogenic Th17 stimuli (IL6 plus IL-1β and IL23) stimuli, there was a significant reduction of IL-17 in the splenocytes from EGR2^-/-^B6/lpr mice compared to control EGR2^fl/fl^B6/lpr mice (**Figures 9G, H**). Nevertheless, EGR2 deletion in B6 mice had no obvious effect on IL-17 production in response to non-pathogenic Th17 stimuli but significantly increased IL-17 production in pathogenic Th17 stimuli activated splenocytes (**Figures 9I, J**). Moreover, we found that EGR2 deletion in B6/lpr mice suppressed naïve CD4^+^ T cell differentiation into Th17 cells but promoted naïve CD4^+^ T cell differentiation into Th1 cells (**Figure 9K**). Together, our data clearly demonstrate that in B6/lpr mice EGR2 has a suppressive role in the *in vitro* induction of IFNγ and Th1 differentiation but a positive regulatory role in *in vitro* induction of IL-17 and Th17 differentiation. However, in B6 mice, EGR2 has a similar suppressive role in the *in vitro* induction of both IFNγ and IL-17.

### EGR2 Deletion Substantially Reduces B220^+^ Double Negative T Cells in B6/lpr Mice

**Figure 10:**
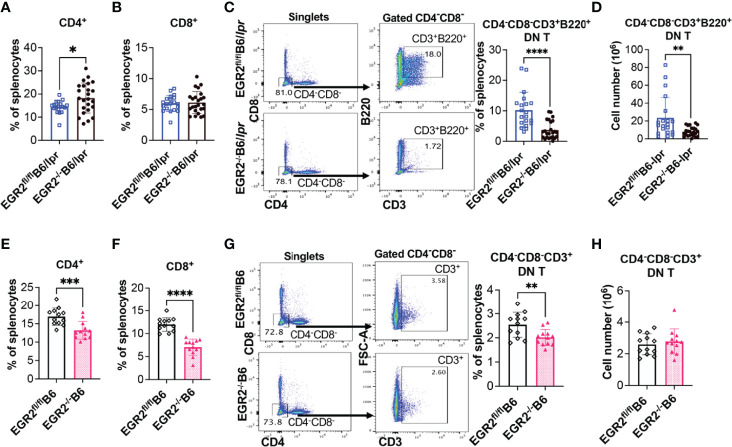
EGR2 deletion in B6/lpr mice dramatically reduces B220^+^ double negative T cells. **(A, B)** Summary graphs show increased percentage of CD4^+^ and no change of CD8^+^ single positive cells in the spleens of EGR2^-/-^B6/lpr mice compared to controls (n≥16). **(C, D)** Representative flow plots and summary graph show that EGR2 deletion significantly reduced the percentage **(C)** and also absolute cell numbers **(D)** of CD4^-^CD8^-^CD3^+^B220^+^ double negative T cells in the spleens of B6/lpr mice. **(E, F)** Summary graphs show decreased percentage of both CD4^+^and CD8^+^ cells in the spleens of EGR2^-/-^B6 mice compared to control EGR2^fl/fl^B6 mice (n=12 each). **(G, H)** Representative flow plots and summary graph show that EGR2 deletion significantly reduced the percentage **(G)**, but not absolute cell numbers **(H)** of CD4^-^CD8^-^CD3^+^ double negative T cells in the spleens of B6 mice. Horizontal bars in the summary graphs indicated mean ± SD. Statistical significance was indicated by asterisks: *p<0.05; **p<0.01; ***p<0.001; ****P<0.0001.

EGR2 deletion in B6/lpr mice decreased IL-17 production in activated splenocytes without suppressing IL-17 expression in the gated CD4^+^ and CD8^+^ T cells (**Figures 9B, C**). We then analyzed the T lymphocyte profiles in EGR2^-/-^B6/lpr and control B6/lpr mice to determine whether EGR2 deletion altered T lymphocyte profiles, leading to the reduction of IL-17 in activated splenocytes. Compared to control EGR2^fl/fl^B6/lpr mice, EGR2^-/-^B6/lpr mice had a significant increase in the percentage of splenic CD4^+^ T, but not CD8^+^ T cells (**Figures 10A, B**). The absolute splenic CD4^+^ and CD8^+^ T cells number were increased in EGR2^-/-^B6/lpr mice (data not shown). In contrast, EGR2 deletion in B6 mice significantly reduced the percentage of both CD4^+^ T and CD8^+^ T cells in the spleens (**Figures 10E, F**). Since EGR2 deletion increased splenic cellularity in B6 mice (**Figure 3C**), the total splenic CD4^+^ T cell number in EGR2^-/-^B6 mice was still comparable to control EGR2^fl/fl^B6 mice (data not shown).

The accumulation of CD4^-^CD8^-^CD3^+^ double negative (DN) T cells is a unique characteristic in *lpr* lupus mice, which is caused by fas*^lpr^* mutation (28–30). The DN T cells identified in *lpr* mice also express B220. Increased DN T cells have also been identified in lupus patients and are involved in lupus pathogenesis (31). Further, DN T cells were major IL-17 producers in lupus. Here, we found that deletion of EGR2 substantially inhibited the development of DN T cells in B6/lpr mice. There was a significant reduction in the B220^+^ DN T cell percentage and also absolute number in the spleens of EGR2^-/-^B6/lpr mice compared to control EGR2^fl/fl^B6/lpr mice (**Figures 10C, D**). The percentage of CD4^-^CD8^-^CD3^+^ DN T cells was low in the spleens of normal B6 mice (around 2.5%). EGR2 deletion reduced the percentage of DN T cells in B6 mice (**Figure 10G**). Due to increased splenic cellularity in EGR2^-/-^B6 mice, there was no difference in the absolute DN T cells number between EGR2^-/-^B6 mice and control EGR2^fl/fl^B6 mice (**Figure 10H**). Together, our data show that EGR2 deletion differentially alters the CD4^+^ and CD8^+^ T cell profiles in the spleens of B6/lpr and B6 mice. Importantly, we found that EGR2 deletion significantly reduced B220^+^DN T cells numbers in B6/lpr mice, which may contribute to reduced IL-17 production in activated splenocytes from EGR2^-/-^B6/lpr mice.

## Discussion

Increased EGR2 expression, rather than decreased EGR2 expression, has been identified in human patients with lupus or systemic sclerosis and the murine models of these two diseases (15, 16). These data seem to contradict the previous reports of the autoimmune suppressive role of EGR2 in B6 mice (10, 11, 13, 27). Moreover, there are inconsistent data with regard to the role of EGR2 in the regulation of Th1/Th17 differentiation and effector T cell response to the infection of different viral pathogens (6, 32–36). Therefore, we believe that it is important to investigate the immune and autoimmune regulatory role of EGR2 in specific pathogenic/autoinflammatory settings. This is the first study to characterize and compare the immune regulatory role of EGR2 *in vivo* in the context of autoinflammation (B6/lpr) versus normal physiology (B6) conditions. Analysis of CD2 specific EGR2^-/-^B6/lpr and EGR2^-/-^B6 mice in this study revealed that while EGR2 has some similar immunological effects in B6/lpr and B6 mice, the differential immune regulatory roles of EGR2 in these two strains were also evident (Summarized in **Table 1**).

**Table 1:** Summary of the similar and differential outcomes of EGR2 deletion in B6/lpr versus B6 mice.

Target	EGR2 deletion in B6/lpr^*^	EGR2 deletion in B6^**^
**Spleen**		
Spleen Weight	Increase	Increase
Splenocytes Count	NC	Increase
**Serum autoantibodies**		
Anti-dsDNA	Decrease	Increase
**Serum immunoglobulin isotypes**		
Total IgG	Decrease	NC
Total IgM	Decrease	NC
IgG1	Decrease	NC
IgG2a	Decrease	NC
**Renal Histopathology**		
IgG Deposition	NC	ND
C3 Deposition	Increased	ND
Large-Caliber Inflammation	Increased	ND
Glomerulus Inflammation	NC	ND
**Cytokines (*In vitro* in activated splenocytes)**		
IFNγ	Increase	Increase
IL-17	Decrease	Increase
**T Cell Activation/Function**		
CD44^+^/CD4^+^ cells	Increase	Increase
CD44^+^/CD8^+^ cells	Trend of Increase	Increase
IFNγ^+^/CD4^+^ cells	Increase	Increase
IFNγ^+^/CD8^+^ cells	NC	Increase
IL17^+^/CD4^+^ cells	NC	Trend of Increase
IL17^+^/CD8^+^ cells	NC	NC
**Th Differentiation**		
Th1	Increase	N/A
Th17	Decrease	N/A
**Immune Cell Proportion (Spleen)**		
GC B cells	Increase	Increase
Plasma Cells	Decrease	NC
LAG3^+^ Treg	NC	Increase
Foxp3^+^ Treg	Decrease	Trend of Increase
DN T Cells	Decrease	Decrease

**NC**, No Change; **ND**, Not Detected; **N/A**, Data Not Available.

^*^ EGR2^-/-^B6lpr mice were compared to EGR2^fl/fl^B6/lpr.

^**^ EGR2^-/-^B6 mice were compared to EGR2^fl/fl^B6 mice.

The most striking finding in this study is that EGR2 deletion in B6/lpr mice significantly suppressed the production of anti-dsDNA autoantibody (**Figure 1E**), a hallmark of the autoimmune parameter in B6/*lpr* mice. Further, EGR2 deletion did not deteriorate renal function in B6/lpr and it only increased inflammation at large caliber vessels (**Figure 2**). These data are inconsistent with the previous finding that EGR2 deletion in B6 mice induced lupus-like autoimmune disease with the development of glomerulonephritis and high levels of anti-dsDNA autoantibodies in serum (10). Consistent with the previous reports (10, 11), we demonstrated that EGR2 deletion in B6 mice promoted splenomegaly, T cell activation, IFNγ and IL-17 production, and GC B development. However, we did not observe a clinical manifestation of lupus-like disease in our EGR2^-/-^B6 mice. The serum levels of anti-dsDNA autoantibodies were still very low in 9-10-months old EGR2^-/-^B6 mice, although they were higher than the littermate control EGR2^fl/fl^B6 mice (**Figure 3**). The lack of apparent autoimmune disease manifestation in our EGR2^-/-^B6 mice may be caused by the differences in the diet, housing condition, and local environment in our animal facility from the others. The influences of commercial diet and animal housing facilities on the disease manifestation in the murine lupus models have been previously reported (23, 37). In addition, due to the overlapping role of EGR2 and EGR3, deletion of both EGR2 and EGR3 genes is necessary for the development of severe lupus-like autoimmune disease in B6 mice (11, 12). We were also unable to detect obvious production of proteinuria in either EGR2^-/-^B6/lpr or control B6/lpr mice. It is likely because although B6/lpr mice produce a significant amount of anti-dsDNA autoantibodies, they only show limited glomerular lesions and do not develop severe nephritis or proteinuria compared to MRL/lpr mice (17, 38). In addition, in the current study, we used the Chemistrip-2GP test strip, a semi-quantitative method to rapidly measure the total protein level in urine. A more sensitive and quantitative method will be considered in future studies for detecting protein levels, especially albumin levels in urine samples.

EGR2 deletion promoted GC response and antibody production in B6 mice due to the loss of immune suppressive function of LAG3^+^ Tregs (11, 12). Here, we demonstrated that EGR2 deletion similarly promoted GC B cell development in B6 and B6/lpr mice (**Figures 4A**, **5A**). Nevertheless, EGR2 deletion significantly suppressed the differentiation of major antibody-secreting cells, CD19^+^CD138^+^ plasmablasts and CD19^-^CD138^+^ plasma cells in the B6/lpr mice but had no obvious effect on plasma cell differentiation in B6 mice (**Figures 4B**, **5B**). Although the mechanism underlying EGR2 differentially regulating plasma cell differentiation in B6/lpr and B6 mice remains unknown, this data provides a cellular mechanism underlying the reduced anti-dsDNA and total immunoglobulins in EGR2^-/-^B6/lpr mice.

Recent studies suggested that atypical Foxp3 independent LAG3^+^ Tregs, other than Foxp3^+^ Tregs, play an important role in suppressing GC response and humoral responses in B6 mice (11, 12, 27). However, it is unlikely that EGR2 regulates inflammation and humoral response in B6/lpr *via* regulating LAG3^+^ Tregs since LAG3^+^ Tregs are already dysfunctional in B6/lpr mice due to the *fas* mutation. A previous study has shown that although the development of LAG3^+^ Tregs is EGR2 and Fas independent, the autoimmune suppressive function of LAG3^+^ Tregs is EGR2 and Fas dependent (11). The adoptive transfer of LAG3^+^ Tregs from Fas-sufficient MRL mice, but not LAG3^+^ Tregs from *fas*-mutant MRL/lpr mice suppressed lupus disease development in recipient MRL/lpr mice (11). In addition, we found that EGR2 deletion significantly reduced the percentage of CD25^+^Foxp3^+^ cells in gated splenic CD4^+^ T cells of B6/lpr mice, but not of B6 mice. It is noteworthy that EGR2 deletion did not change the percentage of CD4^+^CD25^+^Foxp3^+^ Tregs in the spleens of EGR2^-/-^B6/lpr mice due to the increase of the percentage of splenic CD4^+^ T cells (data not shown). Whether EGR2 deletion affects the immune suppressive function of CD25^+^Foxp3^+^Tregs is unknown and warrants further investigation for understanding the differential role of EGR2 in B6/lpr and B6 mice.

Accumulation of CD44^+^ effector T cells and heightened production of IFNγ and IL-17 also play a major role in the development of lupus-like autoimmune disease in EGR2^-/-^B6 mice (10). EGR2 deletion significantly promoted IFNγ production in activated splenocytes from either B6/lpr or B6 mice (**Figure 7**). Due to the autoactivation of T cells in the autoimmune-prone B6/lpr mice, the promotion effect of EGR2 deletion on T cell activation in B6/lpr mice was not as prominent as that in B6 mice (**Figure 8**). There was increased CD44 expression in gated CD4^+^ T cells, but not CD8^+^ T cells from EGR2^-/-^B6/lpr mice. This may explain the increase of IFNγ expression in gated CD4^+^ T cells, but not in gated CD8^+^ T cells of EGR2^-/-^B6/lpr mice (**Figures 7B, C**).

Importantly, we found that EGR2 has an opposite role in the regulation of proinflammatory cytokine IL-17 production in B6/lpr versus B6 mice. Deleting EGR2 *in vivo* significantly suppressed IL-17 production in activated splenocytes from B6/lpr mice but increased IL-17 production in activated splenocytes from B6 mice (**Figure 9**). Intracellular cytokine analysis showed that EGR2 deletion did not affect IL-17 expression in either gated CD4^+^ T cells or CD8^+^ T cells of B6/lpr mice. So, it is likely that EGR2 deletion in B6/lpr mice may reduce immune cell subsets that produce IL-17, such as CD4^+^ and CD8^+^ T cells. However, there were increased CD4^+^ T cell percentage and no change in CD8^+^ T cell percentage in EGR2^-/-^B6/lpr compared to control (**Figures 10A, B**). Importantly, we found that B220^+^ DN T cells were dramatically reduced in EGR2^-/-^B6/lpr mice. Increased DN T cell numbers have been identified in human SLE patients and murine lupus, which correlate with disease activity (31, 39). DN T cells are the primary producer of IL-17 in lupus. The infiltration of IL-17 expressing DN T cells plays a significant role in kidney inflammation and lupus nephritis (31, 39, 40). In addition, DN T cells have also been shown to promote tissue damage by helping B cells to promote the production of IgG and pathogenic anti-dsDNA autoantibodies (41–43). The significantly reduced DN T cells in EGR2^-/-^B6/lpr explains, at least in part, the reduction of IL-17, total immunoglobins, and anti-dsDNA autoantibodies in EGR2^-/-^B6/lpr mice. While B6/lpr strain does not typically show marked gender differences in disease development, we observed that female EGR2^fl/fl^B6/lpr mice had a higher serum level of anti-dsDNA and a higher percentage of B220^+^ DN T cells than male EGR2^fl/fl^B6/lpr mice. Accompanied by these, there was also a more pronounced suppression effect of EGR2 deletion on anti-dsDNA and DN T cells in female B6/lpr mice relative to male B6/lpr mice (**Supplementary Figures 3A, B**). We also noticed that EGR2 deletion had a more substantial effect on spleen weight in female B6/lpr mice relative to male mice (**Supplementary Figure 3C**). However, EGR2 deletion in B6 mice only showed a female-biased effect on spleen weight, but not on serum anti-dsDNA and DN T cells (**Supplementary Figures 3D–F**).

Previous studies have shown that deletion of IL-23R in B6/lpr and MRL/lpr mice reduced DN T, IFNγ- and IL-17- producing cells, suppressed anti-dsDNA autoantibodies and total IgG production, reduced immune complex deposition, and ameliorated lupus nephritis in the *lpr* lupus mice (44, 45). Even though EGR2 deletion significantly reduced DN T cells and suppressed the production of IL-17, anti-dsDNA autoantibody, and total IgG, we did not observe the amelioration of renal inflammation in EGR2^-/-^B6/lpr mice. Indeed, there was even an increase of C3 immunocomplex deposition and inflammation in large-caliber vessels in kidneys of EGR2^-/-^B6/lpr mice. The loss of the mitigation effect on renal inflammation in EGR2^-/-^B6/lpr mice is likely due to enhanced IFNγ production, another key inflammatory cytokine driving renal inflammation and lupus nephritis in *lpr* mice. Given the important role of IL-23/IL-23 receptor signaling pathway in developing and maintaining DN T cells in secondary lymphoid organs (44–46), further investigation of whether EGR2 regulates IL-23/IL23R signaling may aid us to elucidate the mechanism underlying the positive role of EGR2 in regulating DN T cells.

We have shown that EGR2, a negative regulator of T cell activation, was increased in lupus cells (16). Similar phenomena were observed for other genes, such as protein tyrosine phosphatase non-receptor type 22 (PTPN22). PTPN22, a negative regulator of T cell activation, has been associated with the development of multiple autoimmune diseases, including SLE, rheumatoid arthritis, type 1 diabetes, and autoimmune thyroid disease (47). Similar to EGR2, while deletion of PTPN22 in B6 mice resulted in the increased activation of effector T cells and elevated GC response and antibody production, PTPN22 expression/function is increased in SLE (47, 48). Together, our data strongly suggest that the immune and autoimmune regulatory role of EGR2 in normal physiological and different pathological conditions should not be generalized. To fully understand the autoimmune regulatory role (either protective or detrimental) of EGR2, in addition to normal B6 and autoimmune-prone B6/lpr mice, further studies with different autoimmune disease models and cell-specific deletion of EGR2 are warranted.

## Data Availability Statement

The original contributions presented in the study are included in the article/supplementary material. Further inquiries can be directed to the corresponding authors.

## Ethics Statement

The animal study was reviewed and approved by The Institutional Animal Care and Use Committee (IACUC) of Virginia Tech.

## Author Contributions

RD, SA, and CR conceived the study and secured the fund for research. RD designed and performed experiments with assistance from ZW, BH, and KE. RD and SA drafted, wrote, and finalized the manuscript. CR critically reviewed and edited manuscript. All authors have read and approved final manuscript for publication.

## Funding

This study was supported by the Edward Via College of Osteopathic Medicine (VCOM)-Virginia-Maryland College of Veterinary Medicine (VMCVM) One health Research Seed grant (Reference# 10363 PV2C3TH6). The article processing charges (APC) is partially funded by VT Open Access Subvention Fund.

## Conflict of Interest

The authors declare that the research was conducted in the absence of any commercial or financial relationships that could be construed as a potential conflict of interest.

## Publisher’s Note

All claims expressed in this article are solely those of the authors and do not necessarily represent those of their affiliated organizations, or those of the publisher, the editors and the reviewers. Any product that may be evaluated in this article, or claim that may be made by its manufacturer, is not guaranteed or endorsed by the publisher.
