# Supplementary material for: EGR2 Deletion Suppresses Anti-DsDNA Autoantibody and IL-17 Production in Autoimmune-Prone B6/lpr Mice: A Differential Immune Regulatory Role of EGR2 in B6/lpr Versus Normal B6 Mice
Source: Front Immunol. 2022 Jun 15;13:917866. doi: 10.3389/fimmu.2022.917866 (PMC9241489; doi:10.3389/fimmu.2022.917866)
Supplement: Supplementary file 1 [file DataSheet_1.docx]

Supplementary Material


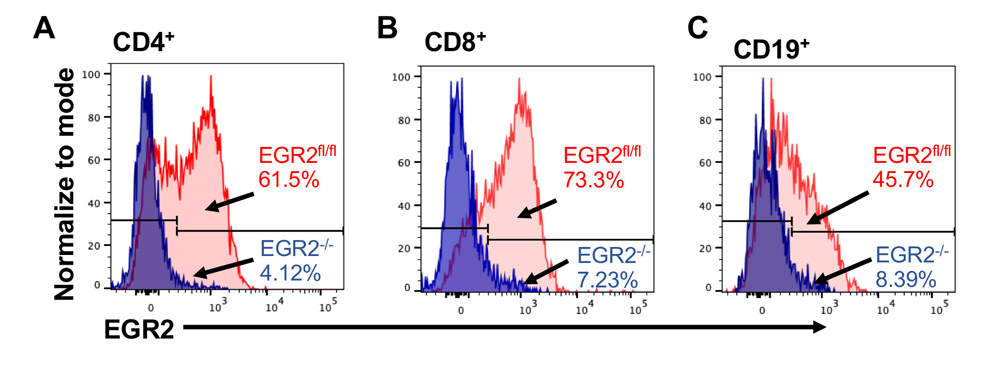


**Supplementary Figure 1.** **Validation of the deletion of EGR2 in lymphocytes of EGR2^-/-^B6/lpr mice.** The splenocytes from EGR2^-/-^B6/lpr and littermate control EGR2^fl/fl^B6/lpr mice were stimulated with PMA plus ionomycin for 5 hrs. The expression of EGR2 in CD4^+^ T, CD8^+^ T, and CD19^+^B cells was determined by intracellular flow stain with PE conjugated anti-EGR2 antibody following the staining of cell surface markers CD4, CD8, and CD19. (**A-C**) Representative flow histograms from at least four independent experiments show the reduced percentage of EGR2 expressing cells and EGR2 expression intensity in the gated splenic CD4^+^ T (A), CD8^+^ T (B), and CD19^+^ B (C) cells of EGR2^-/-^B6/lpr (blue) and control (red) mice.


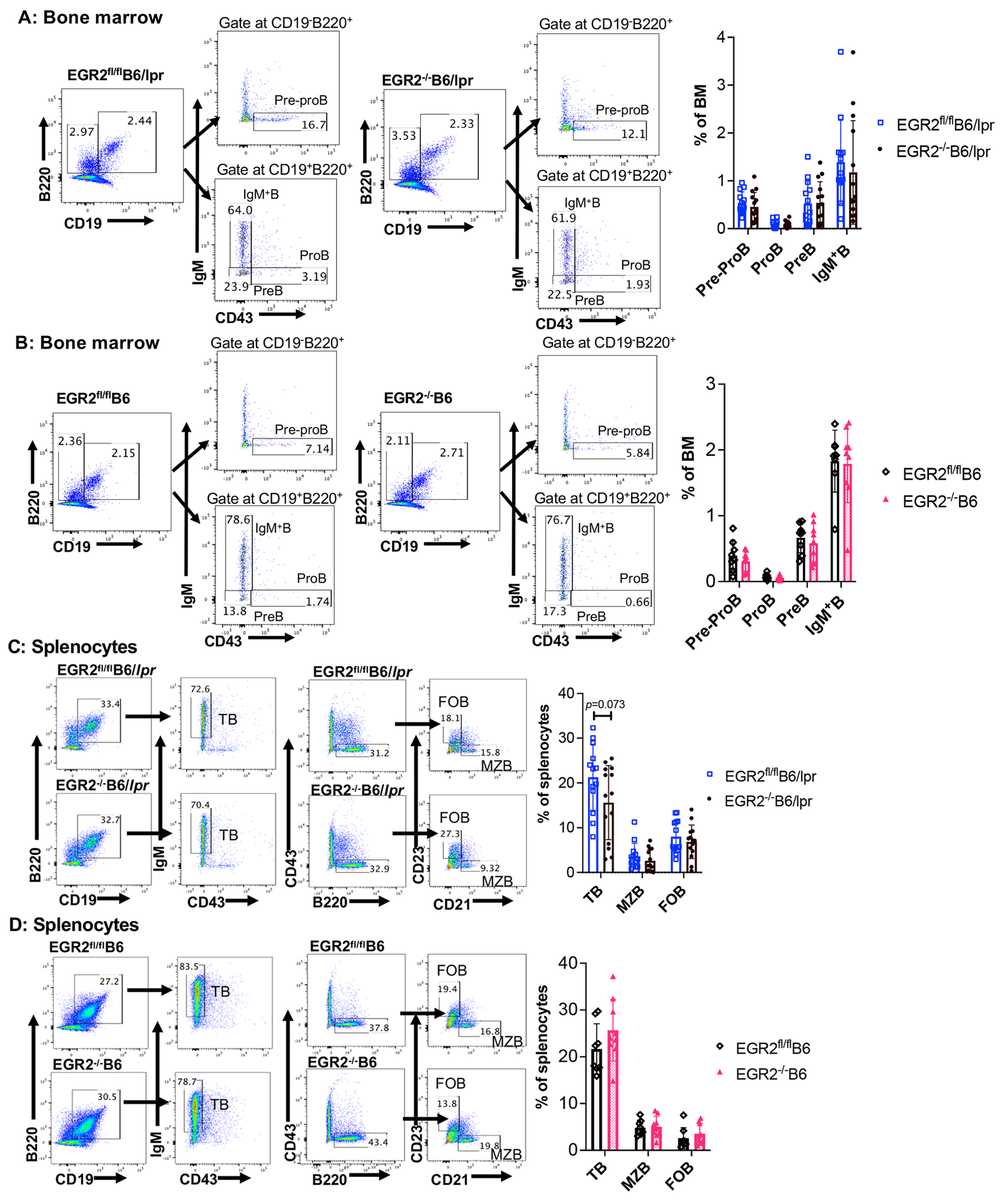


**Supplementary Figure 2. EGR2 deletion has no significant effect on early B cell development in B6/lpr and B6 mice. (A&B)** Representing flow plots and summary graphs show that EGR2 deletion had no obvious effect on the early B cell development in the bone marrows of B6/lpr (A) and B6 (B) mice. The bone marrow cells from EGR2^-/-^B6/lpr and control EGR2^fl/fl^B6/lpr (n≥13), EGR2^-/-^B6 and control EGR2^fl/fl^B6 mice (n≥8) were stained with different surface markers and gated for different B cell precursor subpopulations. Pre-ProB cells were identified as CD19^-^B220^+^CD43^+^IgM^-^; Pro-B cells were defined as CD19^+^B220^+^CD43^+^IgM^-^; Pre-B cells were defined as CD19^+^B220^+^CD43^-^IgM^-^; IgM^+^B (immature and mature B) were defined as CD19^+^B220^+^CD43^-^IgM^+^. (**C&D)** Representing flow plots and summary graphs show that EGR2 deletion had no obvious effect on B cell maturation and differentiation into marginal zone B (MZB) and follicular B (FOB) cells in the spleens of B6/lpr (C) and B6 (D) mice. The splenocytes from EGR2^-/-^B6/lpr and control EGR2^fl/fl^B6/lpr (n≥13), EGR2^-/-^B6 and control EGR2^fl/fl^B6 (n≥8) mice were stained with different surface markers and gated for transitional B (TB) cells, MZB, and FOB. TB cells were identified as CD19^+^B220^+^CD43^-^IgM^+^; MZB cells were defined as B220^+^CD43^-^CD21^+^CD23^-^ ; FOB cells were defined as B220^+^CD43^-^CD21^-^CD23^+^. Horizontal bars in the summary graphs indicated mean ± SD.

**
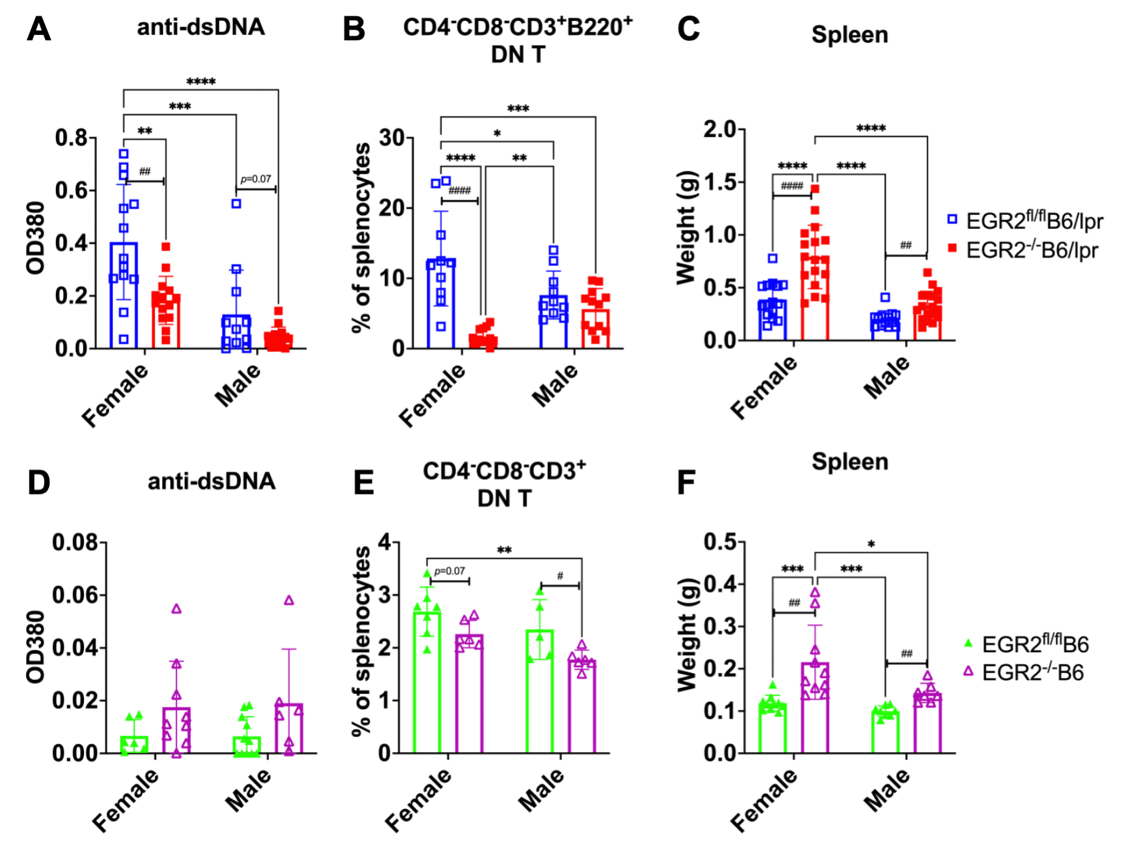
**

**Supplementary Figure 3. EGR2 deletion has a more pronounced effect on select immune parameters in female B6/lpr compared to male B6/lpr mice.** (**A-C**) Summary graphs show the female-biased effect of EGR2 deletion on anti-dsDNA (A), CD4^-^CD8^-^CD3^+^B220^+^ double-negative T cells (B) and spleen weight (C) in B6/lpr mice. (**D-E**) Summary graphs show the female biased effect of EGR2 deletion only on spleen weight (F), but not on anti-dsDNA (D) and CD4^-^CD8^-^CD3^+^double-negative T cells (E) in B6 mice. Horizontal bars in the summary graphs indicated mean ± SD (n ≥ 5). Two-way ANOVA with Tukey’s multiple comparison tests were performed to assess the statistical significance among different gender and genotype groups. The groups have significant difference in the means were connected and indicated a by asterisk (*, *p*<0.05; **, *p*<0.01; ***, *p*<0.001; ****, *p*<0.0001). Unpaired student t tests were performed to evaluate the statistical significance between control and knockout mice in the same gender and the same genetic background. The statistical significance was depicted with number sign *(#*, *p*<0.05; ##, *p*<0.01; ####, *P*<0.0001).
